# Validation of the Croatian Versions of DASH, PRWE and Mayo Wrist Score in Patients with Distal Radius Fractures

**DOI:** 10.3390/jcm14227924

**Published:** 2025-11-08

**Authors:** Borjan Josifovski, Matej Pedisic, Mirela Vuckovic, Ksenija Bazdaric, Zdravko Jotanovic

**Affiliations:** 1 University Hospital for Orthopaedics and Traumatology Lovran, Marsala Tita Promenade 1, 51415 Lovran, Croatiamatej.pedisic@gmail.com (M.P.);; 2 Department of Physiotherapy, Faculty of Health Studies, University of Rijeka, Viktora Cara Emina 5, 51000 Rijeka, Croatia; 3 Department of Basic Medical Sciences, Faculty of Health Studies, University of Rijeka, Viktora Cara Emina 5, 51000 Rijeka, Croatia; 4 Department of Orthopedics and Physical Medicine, Faculty of Medicine, University of Rijeka, Branchetta Brothers 20, 51000 Rijeka, Croatia

**Keywords:** DASH, distal radius fractures, Croatian version, Mayo Wrist Score, PRWE, validation

## Abstract

**Background/Objectives**: Distal radius fractures are common upper extremity injuries requiring reliable outcome measures for accurate clinical assessment. This study aimed to validate the Croatian versions of the Disabilities of the Arm, Shoulder and Hand (DASH), Patient-Rated Wrist Evaluation (PRWE), and Mayo Wrist Score (MWS) in patients with distal radius fractures. **Methods**: DASH, PRWE, and MWS Croatian versions were evaluated in 128 patients using standardized translation, cultural adaptation, factor analysis, and internal consistency (Cronbach’s α). **Results**: PRWE-Cro and DASH-Cro demonstrated excellent validity and internal consistency (α > 0.95). QuickDASH-Cro showed high reliability and is recommended as a practical alternative. MWS was validated for the first time, showing good validity and acceptable internal consistency (α = 0.71). **Conclusions**: PRWE-Cro and DASH-Cro are validated, reliable instruments suitable for both clinical practice and research in Croatian-speaking populations while MWS can be used as a quick screening tool.

## 1. Introduction

Distal radius fractures (DRFs) are among the most common fractures, accounting for 17.5% of all adult fractures [1]. Functional outcomes following treatment of these injuries can vary widely, and objective clinical assessments—such as range of motion, grip strength, and radiographic measurements—often fail to capture the patient’s perspective on pain, disability, and return to function. This highlights the need for validated questionnaires that comprehensively integrate clinical examination findings with assessments of activities of daily living. In routine clinical practice, it is essential that the selected questionnaire be simple and understandable for patients, and time-efficient, reliable, and suitable for use in scientific research. Consequently, the use of patient-reported outcome measures (PROMs) has become essential in both clinical practice and research for evaluating treatment effectiveness and guiding patient-centered care [2]. PROMs provide valuable insights into patients’ perceptions of symptoms, functional limitations, and overall quality of life [2].

Over the past two decades, PROMs for the functional assessment of the upper extremity have been increasingly developed, aiming to objectify the patient’s clinical status and enable functional monitoring over time [3]. For hand and wrist conditions, a total of 22 specific PROMs have been identified in the literature [4]. Among these, the most commonly used are the Disabilities of the Arm, Shoulder and Hand (DASH) questionnaire, the Patient-Rated Wrist (and Hand) Evaluation [PRW(H)E], and the QuickDASH [4]. These PROMs are most frequently applied in studies focusing on traumatic hand and wrist conditions [4]. A recent study demonstrated that using the QuickDASH and PRWHE alongside the Mayo Wrist Score (MWS) is a critical approach for assessing pain and functional outcomes in patients with distal radius fractures [5].

The DASH is a region-specific instrument consisting of 30 items that assess disability and symptoms in the upper extremity across a wide range of conditions, and it is among the most frequently used outcome tools in orthopedic research and practice [6,7]. The QuickDASH is a shortened version of the DASH (11 items), designed to measure physical function and symptoms in patients with any musculoskeletal disorder of the upper limb [8,9]. The PRWE is a joint-specific instrument with 15 items focused on wrist pain and function, and it has demonstrated excellent responsiveness in patients with distal radius fractures [10]. The MWS is a brief, 4-item instrument incorporating both clinician and patient input, and it is widely used as a PROM in wrist trauma studies due to its ease of application in clinical settings [11,12]. Nevertheless, to the best of our knowledge, MWS has not been formally validated. The existing literature critiques its mixed subjective–objective format and highlights the lack of established reliability and validity compared to other PROMs such as the PRWE and DASH [12].

Despite their widespread use and proven utility in English-speaking populations, the applicability of PROMs across different languages and cultural settings requires systematic translation and validation procedures [13]. Without cultural adaptation, direct translations may compromise content validity and result in inaccurate assessments. The lack of validated Croatian versions of the DASH, QuickDASH, PRWE, and MWS has limited their routine use in clinical practice and research among Croatian-speaking populations.

This study aimed to translate and culturally adapt the DASH, QuickDASH, PRWE, and MWS into the Croatian language and to evaluate their psychometric properties—specifically validity and reliability—in patients with distal radius fractures.

## 2. Materials and Methods

### 2.1. Study Design and Participants

A cross-sectional clinimetric study was conducted from 30 January 2024 to 28 February 2025 at the University Hospital for Orthopaedics and Traumatology in Lovran, Croatia and the Kantrida Nursing Home in Rijeka, Croatia.

A total of 128 patients with DRFs completed PROMs. Both conservatively and surgically treated patients were included.

### 2.2. Inclusion and Exclusion Criteria

The study included patients aged 18 to 90 years, regardless of gender, who had sustained a distal radius fracture at least three months prior to enrollment. Distal radius fractures were selected as the target condition because they are among the most common upper extremity injuries and affect both wrist and hand function, making them ideal for testing region-specific instruments such as the DASH, PRWE, and Mayo Wrist Score. The 3-month post-injury time point was chosen because, by that stage, most patients have completed treatment and early rehabilitation and reached a stable functional status, enabling reliable evaluation of pain and disability while still reflecting the consequences of the fracture. Exclusion criteria were the following: age under 18 or over 90 years, incomplete questionnaire responses, less than three months between the fracture and study enrollment, cognitive impairment, legal guardianship, or insufficient understanding of the Croatian language.

### 2.3. Procedure

The first group of participants (n = 115) was enrolled during follow-up visits at the University Hospital for Orthopaedics and Traumatology in Lovran, Croatia. The second group (n = 13) consisted of residents of the Kantrida Nursing Home for the Elderly in Rijeka, Croatia, who had sustained distal radius fractures and met the study criteria. These individuals were approached directly at the care facility with the cooperation of healthcare staff. Only participants from both groups who met the inclusion criteria were enrolled in the study. All participants received a detailed explanation of the study’s purpose, and written informed consent was obtained. They were then invited to complete the questionnaires, with authors BJ and MP present to provide clarification and ensure completeness. This supervised administration helped reduce misunderstandings and minimize missing data.

### 2.4. Translation and Adaptation

The cross-cultural adaptation of the PRWE, DASH, and MWS questionnaires was performed according to established guidelines for translation and validation [13]. The process began with the forward translation of the original questionnaires into Croatian, independently carried out by two bilingual translators whose native language is Croatian. Discrepancies between the translations were resolved through discussion and consensus in collaboration with two orthopedic surgeons.

The agreed-upon Croatian versions were then back-translated into English by two independent native English speakers, both blinded to the original versions. A review panel—comprising one of the initial translators, one of the back-translators, a methodology expert in outcome measures, a physiotherapist, and two orthopedic surgeons (one specializing in hand and wrist surgery)—evaluated all versions. Any inconsistencies identified during the review were resolved by consensus.

A pre-final version of each questionnaire was then developed and pilot-tested on a sample of five patients with distal radius fractures. Based on their feedback, minor adjustments were made, resulting in the final Croatian versions of the PRWE (PRWE-Cro), DASH (DASH-Cro), and MWS (MWS-Cro).

### 2.5. Instruments

#### 2.5.1. Disabilities of the Arm, Shoulder and Hand Questionnaire (DASH)

The Disabilities of the Arm, Shoulder and Hand (DASH) questionnaire, developed in 1996 by the Upper Extremity Collaborative Group (UECG) [6], was created to address the need for a standardized, region-specific instrument for evaluating upper extremity disability across a wide range of musculoskeletal conditions. The development process involved expert consensus, patient input, and psychometric validation to ensure clinical relevance, reliability, and sensitivity to change across diverse patient populations and treatment modalities. The DASH consists of 30 items grouped into two domains: (1) activity-related difficulty (23 items), which evaluates the extent to which patients experienced difficulty performing specific daily activities during the past week; and (2) symptom severity and social and occupational limitations (7 items), which assess pain, paresthesia, and sleep disturbances, as well as work- and social-functioning restrictions during the same period. Scores range from 0 to 100, with 0 indicating no disability and 100 representing the most severe functional impairment of the upper extremity.

#### 2.5.2. Quick Disabilities of the Arm, Shoulder and Hand (QuickDASH) Questionnaire

The Quick Disabilities of the Arm, Shoulder and Hand (QuickDASH) questionnaire is a shortened version of the original DASH instrument, developed by the Upper Extremity Collaborative Group to provide a more practical and time-efficient tool for assessing upper extremity disability [8]. It was designed to reduce respondent burden while retaining the measurement precision and psychometric robustness of the full DASH. The QuickDASH consists of 11 items selected through rigorous item-reduction procedures based on reliability and factor analysis. These items evaluate the degree of difficulty experienced in performing daily physical activities and the severity of symptoms such as pain and tingling in the arm, shoulder, or hand during the preceding week. Each item is scored on a 5-point Likert scale ranging from 1 (no difficulty or symptoms) to 5 (unable to perform or extreme symptoms). The total QuickDASH score is calculated by summing the completed item scores, computing their mean, subtracting 1, and multiplying by 25, yielding a standardized score from 0 (no disability) to 100 (most severe disability).

#### 2.5.3. Patient-Rated Wrist Evaluation Questionnaire (PRWE)

The Patient-Rated Wrist Evaluation (PRWE) questionnaire is a highly reliable and valid instrument, originally developed in 1996 [10]. It was designed to address the need for a concise, patient-centered tool specifically targeting wrist pain and functional disability following upper-extremity injuries such as distal radius fractures. The PRWE consists of 15 items divided into two subscales: pain (5 items) and function (10 items). Each item is scored from 0 to 10, where higher values indicate greater pain or disability. The pain subscale score is calculated as the sum of its five items (maximum 50), while the function subscale score (maximum 100) is obtained by summing its 10 items and dividing by two to place it on the same 0–50 scale as the pain subscale. The total PRWE score is then derived by adding the two subscale scores, yielding a final score ranging from 0 (no pain or disability) to 100 (maximum pain and disability).

#### 2.5.4. Mayo Wrist Score (MWS)

In 1987, Cooney et al. [11] analyzed wrist fractures using a modification of the Green and O’Brien score, revising the demerit criteria and omitting radiographic indices; this approach later formed the basis of what became known as the Mayo Wrist Score (MWS; Mayo Clinic, Rochester, MN, USA) [12]. The MWS is a clinician-administered tool that combines subjective and objective measures to quantify wrist function following trauma or disease. It has been widely used due to its simplicity and its ability to capture both patient-reported symptoms and physical examination findings. The MWS assesses wrist function using four domains: pain intensity (0, 15, 20, or 25 points); functional status—specifically, the ability to return to regular work (0, 15, 20, or 25 points); active range of motion—measuring wrist flexion and extension compared to the contralateral side (0, 5, 10, 15, or 25 points); and grip strength—comparing grip strength of the affected hand to the contralateral side (0, 5, 10, 15, or 25 points). The total possible score ranges from 0 to 100 points and is interpreted as follows: 90–100 points indicate an excellent result; 80–90, a good result; 60–80, a satisfactory result; and below 60, a poor result.

### 2.6. Ethics

Ethics approval was obtained from the University Hospital for Orthopaedics and Traumatology in Lovran, Croatia (reference number 02-249/2021), and the Faculty of Medicine at the University of Rijeka, Croatia ((CLASS: 007-08/22-01/25, URNUMBER: 2170-24-04-3/1-22-6)) and authorization was obtained from the Kantrida Nursing Home in Rijeka, Croatia (reference number 01-3972/25).

Written informed consent was obtained from all participants prior to study commencement.

All collected data were anonymized and stored in a secure database for subsequent statistical analysis.

### 2.7. Statistical Analysis

Categorical data are shown by frequency and relative frequency. Quantitative data are presented as mean and standard deviation. Average values of variables that have non-normal distribution (tested with the Kolmogorov–Smirnov test) are presented with median and interquartile range.

Before factor analysis, we have transformed the values on MWS to z-scores as the items responses have 2 scales (items 1–2 have a 4-point scale and items 3–4 have a 5-point scale). The transformed values were used for the factor analysis and reliability analysis.

We performed an exploratory factor analysis (EFA) and a Principal Axis Factoring extraction with oblimin rotation to examine the underlying structure of the measured constructs. Before factor analysis, we ran the Kaiser–Meyer–Olkin (KMO) measure of sampling adequacy and Bartlett’s test of sphericity to test the suitability of the item correlation matrix for factoring. We included the extracted factors with the eigenvalue >1, which accounted for more >10% of the variance and which passed visual inspection on the screen plot.

Reliability was assessed with the coefficient of internal consistency Cronbach’s α and interpreted as follows: α > 0.70 as acceptable, α > 0.80 as good, and α > 0.90 as excellent reliability [14].

Correlations were calculated with the Spearman coefficient of correlation, as the distributions did not follow a normal distribution (Shapiro–Wilk test). The strength of the correlations was interpreted according to Schober et al. [15] as follows: negligible (<0.1), weak (0.1–0.39), moderate (0.4–0.69), strong (0.7–0.89), and very strong (≥0.9).

The sample size was determined according to the previous similar studies [16,17,18,19,20,21], and the subject-to-item ratio was 4.2:1.

A *p* < 0.05 value was considered statistically significant. Jamovi version 2.3.28.0. (https://www.jamovi.org; Sydney, Australia) was used for statistical analysis.

## 3. Results

There were 128 participants, and their characteristics are presented in Table 1. Participants were median age 65 years (IQR 54–74), mostly female (73%), right-handed (52%), dominant hand (54%), and treated conservatively (65%).

### 3.1. Reliability Analysis of DASH, QuickDASH, PRWE and MWS

The overall reliability results are presented in Table 2. The detailed tables are presented in Supplementary Materials (Tables S1–S8).

#### 3.1.1. Reliability Analysis of DASH

The internal consistency of the DASH scales is presented in Table 2 and in detail in Supplementary Materials, Tables S1–S4. The internal consistency of total DASH was very high: Cronbach’s α = 0.98 (Table S1). Both DASH factors have very high reliability; DASH activities subscale—α = 0.98 (Table S2) and DASH symptoms—α = 0.92 (Table S3).

#### 3.1.2. Reliability Analysis of QuickDASH

The internal consistency of the Quick-DASH scale was very high; Cronbach’s α = 0.94 (Table S4).

#### 3.1.3. Reliability Analysis of PRWE

Item reliability statistics of PRWE is presented in Table 2 and in detail in Supplementary Materials, Tables S6–S8. The internal consistency of the whole PRWE scale was very high: Cronbach’s α = 0.97 (Table S5). The PRWE function factor had high reliability of 0.97 (Table S6), and PRWE pain also had high reliability of 0.93 (Table S7).

#### 3.1.4. Reliability Analysis of MWS

Item reliability statistics of MWS are presented in Table 2 and in detail in Supplementary Materials, Table S8. The internal consistency of the MWS was acceptable: Cronbach’s α = 0.71 (Table S9).

### 3.2. Construct Validity of DASH, QuickDASH, PRWE and MWS

#### 3.2.1. Construct Validity of DASH Questionnaire

The KMO value (0.94) and Bartlett’s test of sphericity (*p*  <  0.001) indicated that factor analysis is appropriate for these data. Principal Axis Factoring (PAF) analysis yielded a two-factor model with 66% of the construct variance explained [eigen values 18.2 (explained variance 46%) and eigen value 1.3 (explained variance 19%)] (additional scree plot in Figure S1).

The structure matrix (correlations of each item with the extracted dimensions) and the obtained factorial structure of the DASH questionnaire are complex (Table 3).

All items included had high loadings (>0.40), indicating strong relationships between the items and the extracted factor, but items 17, 22 and 23 had factor loadings on both factors. The correlation between the factors is high, r = 0.73, indicating that this solution is not yielding two factors but two facets of the same factor.

#### 3.2.2. Construct Validity of QuickDASH Questionnaire

The factor analysis of QuickDASH scores is presented in Supplementary Materials. The KMO value (0.93) and Bartlett’s test of sphericity (*p*  <  0.001) indicated that factor analysis is appropriate for these data. Principal Axis Factoring (PAF) analysis yielded a one-factor model with 64% of the construct variance explained (eigen value 5.8) (Table S1, additional scree plot in Figure S2). The structure matrix (correlations of each item with the extracted dimensions) and the obtained factorial structure are simple—unidimensional. All included items had high loadings (>0.60).

#### 3.2.3. Construct Validity of PRWE Questionnaire

The KMO value (0.92) and Bartlett’s test of sphericity (*p*  <  0.001) indicated that factor analysis is appropriate for these data. Principal Axis Factoring (PAF) analysis yielded a complex structure, a two-factor model (additional scree plot in Figure S3) with 75% of the construct variance explained (Table 4).

The structure matrix (correlations of each item with the extracted dimensions) and the obtained factorial structure are presented in Table 4, and they correspond to two factors—function and pain. Those two factors are in high correlation (r = 0.71). All items including had high loadings (>0.70) indicating strong relationships between the items and the extracted factors (Table 4).

#### 3.2.4. Construct Validity of MWS

The KMO value (0.68) and Bartlett’s test of sphericity (*p*  <  0.001) indicated that factor analysis is appropriate for the data. Principal Axis Factoring (PAF) analysis yielded a one-factor model with 41% of the construct variance explained (eigen value 1.60) (additional scree plot in Figure S4).

The structure matrix (correlations of each item with the extracted dimensions) and the obtained factorial structure are simple (Table 5). All included items had high loadings (>0.50).

### 3.3. Correlations of Scales

The correlation between MWS, PRWE, and DASH is presented in Table 6.

The MWS is significantly and moderately negatively associated with both PRWE (r_s_ = −0.58; *p* < 0.001) and DASH scales (r_s_ = −0.61; *p* < 0.001). PRWE and DASH scores are significantly and strongly positively correlated (r_s_ = 0.71; *p* < 0.001).

## 4. Discussion

The DASH and PRWE questionnaires are the most commonly used patient-reported outcome measures (PROMs), and numerous studies have confirmed their validity, reliability, and internal consistency for assessing functional outcomes in patients following distal radius fractures [16]. Our study is the first validation of the DASH, PRWE, and MWS in the Croatian language, and to the best of our knowledge, the first validation of MWS [17]. Both DASH-Cro and PRWE-Cro have excellent construct validity and reliability, and those results are in line with previously obtained results of cross-cultural validations [18,19,20,21]. MWS-Cro is also a valid instrument but with the lower validity and reliability, probably because of its shortness (4 items) compared to the DASH (30 items) and PRWE (15 items).

The DASH questionnaire consists of a total of 30 items divided into two components: an activity component (items 1–23) and a symptom component (items 24–30). Factor analysis of the DASH questionnaire identified two factors that together explained 65.8% of the total variance. Most items were clearly clustered within one of the two factors—functional activity and symptoms; however, minor discrepancies were observed with items 22 and 23. Item 22 (During the past week, to what extent has your arm, shoulder or hand problem interfered with your normal social activities with family, friends, neighbors or groups?) showed moderate factor loadings on both factors (0.487 on the functional factor and 0.427 on the symptom factor), suggesting potential overlap between the functional and symptom components in this item. Item 23 (During the past week, were you limited in your work or other regular daily activities as a result of your arm, shoulder or hand problem?), which theoretically belongs to the functional domain, loaded on the symptom factor, possibly indicating a semantic shift that occurred during the translation process of the questionnaire into Croatian. Apart from these two items, all remaining items demonstrated strong inter-item correlations (ranging from 0.575 to 0.943), as shown in Table 2. Kleinlugtenbelt et al. conducted a similar study in the Netherlands and reported good content validity as well as reliable and internally consistent instruments for evaluating patients with distal radius fractures. Unlike our study, which closely aligned with the original construct, Kleinlugtenbelt et al. identified five factors, explaining less of a total variance (58%) [16]. Vucetic et al. reported results similar to ours, with standardized factor loadings that were statistically significant and ranged from 0.54 to 0.85 [22]. Similarly, de Klerk et al. found high factor loadings for their two-factor model, ranging from 0.597 to 0.896 [23].

The QuickDASH is a shorter and faster version of the original DASH questionnaire, often considered more practical and user-friendly by clinicians. Results of our factor analysis indicate very good validity (64% variance and one factor structure) and high reliability of the QuickDASH (Cronbach’s α = 0.93). The resulting factorial structure was simple, with all items showing high factor loadings (>0.60), further supporting that the questionnaire measures one dimension related to the functional status of the upper extremity. These findings suggest that the QuickDASH-Cro possesses a stable and unidimensional factor structure and can be considered a suitable instrument for the functional assessment of the arm, shoulder, and hand in clinical settings.

The validated structure of the PRWE in our study was consistent with the original; the factors analysis yielded a two-factor solution—function and pain. The reliability of PRWE is very high for the whole scale (0.97) as well as the subscales (function: α = 0.97, pain: α = 0.93) that are highly correlated, indicating, in fact, a unidimensional solution (one factor with two facets). PRWE and DASH scores are significantly and strongly positively correlated (r_s_ = 0.71; *p* < 0.001). Similarly, Alfie et al. reported a Cronbach’s α of 0.96, consistent with findings from other studies [24,25,26]. The PRWE (Patient-Rated Wrist Evaluation) was developed as a questionnaire focused solely on the wrist and hand, in contrast to the DASH questionnaire, which assesses the entire upper limb (shoulder, elbow, and hand), and some authors even emphasize its superiority over the DASH, citing that it is faster and easier to administer [27,28].

Validation of the Mayo Wrist Score was performed for the first time, and with factor analysis of the Mayo Wrist Score questionnaire we extracted a single factor, which explained 40.1% of the total variance, indicating lower validity. The reliability of the Mayo Wrist Score was also found to be acceptable, with a Cronbach’s α of 0.71. Although Mayo Wrist Score has shown good but lower validity than DASH and PRWE, we have to take into account that the measure has only four items and conclude that this result is satisfying. The Mayo Wrist Score was significantly and moderately negatively associated with both PRWE (r_s_ = −0.58; *p* < 0.001) and DASH scales (r_s_ = −0.61; *p* < 0.001). We believe that these results do not preclude Mayo Wrist Score usage; on the contrary, from a practical standpoint, the Mayo Wrist Score can be used as a quick and simple screening tool that can later be supplemented by more nuanced instruments like the PRWE and DASH.

### Limitations of the Study

Our study has some limitations; first, only one condition was studied—distal radius fracture without a control group. Second, due to organizational constraints we did not calculate test–retest reliability and instead calculated the internal consistency. Finally, the sample was sufficient for validation but not very large, and larger testing and validations of the Mayo Wrist Score in different languages are needed.

## 5. Conclusions

When selecting the most appropriate questionnaire for assessing the functional status of the hand, it is crucial to balance the psychometric properties of the instrument with its practical applicability. The DASH represents a comprehensive measurement tool that covers the entire upper extremity; however, its relatively lengthy format can be a limitation, especially in situations requiring rapid functional assessment, so we advise using the QuickDASH version in clinical settings.

In contrast, the PRWE is specifically focused on the functional evaluation of the hand. Due to its fewer items, ease of administration, and high reliability and validity, the PRWE has proven to be an exceptionally useful tool for routine use, particularly in patients with localized wrist pathologies.

The Mayo Wrist Score, being the shortest among the instruments mentioned, allows for a quick and basic assessment of hand functional status. Although less comprehensive, its acceptable reliability makes it valuable in everyday clinical practice when time constraints exist or as a complementary tool alongside more complex questionnaires or diagnostic methods.

This is the first validation of the Mayo Wrist Score, and despite its limitations we recommend its usage as a quick screening tool. PRWE-Cro and DASH-Cro are larger instruments, valid and reliable, suitable for both clinical practice and research in Croatian-speaking populations.

## Figures and Tables

**Table 1:** Participants’ characteristics.

Variable	n (%) or C (25–75)
Age	65 (54–74)
Sex	
Male	35 (27)
Female	93 (73)
Hand	
Left	60 (47)
Right	68 (53)
Dominant hand	
Yes	71 (55)
No	57 (45)
Treatment	
Conservative	84 (66)
Operation	44 (34)

**Table 2:** Reliability of the DASH, PRWE, and MWS scales and subscales.

Scale	Reliability Cronbach’s α
DASH	0.98
DASH activities subscale	0.98
DASH symptoms subscale	0.92
QuickDASH	0.94
PRWE	0.97
PRWE function subscale	0.97
PRWE pain subscale	0.93
MWS	0.71

**Table 3:** Structure matrices (correlations of each item with the extracted dimensions) of the DASH factor analysis *.

Item	Factor 1 Activities	Factor 2 Symptoms
DASH_1	0.663	
DASH_2	0.758	
DASH_3	0.900	
DASH_4	0.831	
DASH_5	0.856	
DASH_6	0.678	
DASH_7	0.815	
DASH_8	0.653	
DASH_9	0.742	
DASH_10	0.798	
DASH_11	0.778	
DASH_12	0.924	
DASH_13	0.943	
DASH_14	0.784	
DASH_15	0.750	
DASH_16	0.710	
DASH_17	0.575	
DASH_18	0.584	
DASH_19	0.719	
DASH_20	0.531	
DASH_21	0.835	
DASH_22	0.487	0.427
DASH_23		0.503
DASH_24		0.935
DASH_25		0.847
DASH_26		0.663
DASH_27		0.631
DASH_28		0.707
DASH_29		0.712
DASH_30		0.596
Eigen value	18.23	1.35
% variance	46.3	19.5
Total variance	65.8	

Legend: values <0.40 are not presented; * ‘Principal Axis Factoring’ extraction method was used in combination with an ‘oblimin’ rotation.

**Table 4:** Structure matrices (correlations of each item with the extracted dimensions) of the PRWE factor analysis *.

Item	Factor 1 Function	Factor 2 Pain
PRWE_1		0.746
PRWE_2		0.994
PRWE_3		0.764
PRWE_4		0.826
PRWE_5		0.768
PRWE_6	0.807	
PRWE_7	0.777	
PRWE_8	0.883	
PRWE_9	0.772	
PRWE_10	0.816	
PRWE_11	0.800	
PRWE_12	0.865	
PRWE_13	0.981	
PRWE_14	0.856	
PRWE_15	0.938	
Eigen value	9.14	1.08
% variance	48.9	25.8
Total variance	74.7	

Legend: Values <0.40 are not presented; * ‘Principal Axis Factoring’ extraction method was used in combination with an ‘oblimin’ rotation.

**Table 5:** Structure matrices (correlations of each item with the extracted dimensions) of the MWS factor analysis *.

Item	Factor 1 MWS
Z_MWS_1	0.477
Z_MWS_2	0.511
Z_MWS_3	0.739
Z_MWS_5	0.754
Eigen value	1.60
% total variance	40.1

Legend: * ‘Principal Axis Factoring’ extraction method was used in combination with an ‘oblimin’ rotation.

**Table 6:** Correlation of MWS, PRWE, and DASH.

		MWS_total	PRWE_total
**MWS_total**	r_s_	-	
	df	-	
	*p*-value	-	
**PRWE_total**	r_s_	−0.581	-
	df	124	-
	*p*-value	<0.001	-
**DASH_total**	r_s_	−0.606	0.708
	df	125	125
	*p*-value	<0.001	<0.001

Legend: r_s_—Spearman’s rho; df—degrees of freedom; MWS_total—Mayo Wrist Score total score, PRWE_total PRWE total score, DASH_total—DASH—total score.

## Data Availability

The original contributions presented in this study are included in the article/Supplementary Materials. Further inquiries can be directed to the corresponding author.

## Supplementary Materials

The following supporting information can be downloaded at: https://www.mdpi.com/article/10.3390/jcm14227924/s1, Table S1. DASH total item reliability statistics (Cronbach’s α = 0.97); Table S2. DASH activities subscale item reliability statistics (Cronbach’s α = 0.98); Table S3. DASH symptoms item reliability statistics (Cronbach’s α = 0.92); Table S4. Quick DASH item reliability statistics (Cronbach’s α = 0.94); Table S5. PRWE item reliability statistics (Cronbach’s α = 0.97); Table S6. PRWE factor 1—function item reliability statistics (Cronbach’s α = 0.97); Table S7. PRWE factor 2—pain item reliability statistics (Cronbach’s α = 0.97); Table S8. MWS item reliability statistics (Cronbach’s α = 0.71); Table S9. Structure matrices (correlations of each item with the extracted dimensions) of the quick DASH factor analysis; Figure S1. Scree plot of the DASH factor analysis; Figure S2. Scree plot of the quick-DASH factor analysis; Figure S3. Scree plot of the PRWE factor analysis; Figure S4. Scree plot of the MWS factor analysis.

## Abbreviations

The following abbreviations are used in this manuscript:

DASH	Disabilities of the Arm, Shoulder and Hand questionnaire
DASH-Cro	Croatian version of the DASH
df	Degrees of freedom
DRF	Distal radius fractures
EFA	Exploratory Factor Analysis
IQR	Interquartile Range
KMO	Kaiser–Meyer–Olkin
MWS	Mayo Wrist Score
MWS-Cro	Croatian version of the Mayo Wrist Score
PAF	Principal Axis Factoring
PROMs	Patient-Reported Outcome Measures
PRWE	Patient-Rated Wrist Evaluation questionnaire
PRWE-Cro	Croatian version of the PRWE
PRWHE	Patient-Rated Wrist and Hand Evaluation questionnaire
QuickDASH	Quick Disabilities of the Arm, Shoulder and Hand questionnaire
Spearman’s rho	Spearman’s rank correlation coefficient
UECG	Upper Extremity Collaborative Group
