# Supplementary material for: Validation of the Croatian Versions of DASH, PRWE and Mayo Wrist Score in Patients with Distal Radius Fractures
Source: J Clin Med. 2025 Nov 8;14(22):7924. doi: 10.3390/jcm14227924 (PMC12653344; doi:10.3390/jcm14227924)
Supplement: Supplementary file 1 [file jcm-14-07924-s001.zip › jcm-3963373-supplementary.pdf]

Supplementary Material

Table S1. DASH total item reliability statistics (Cronbach's α=0.97)

	If item dropped
	**Cronbach's α**
DASH 1	0.977
DASH 2	0.977
DASH 3	0.977
DASH 4	0.977
DASH 5	0.977
DASH 6	0.977
DASH 7	0.977
DASH 8	0.977
DASH 9	0.977
DASH 10	0.977
DASH 11	0.977
DASH 12	0.977
DASH 13	0.977
DASH 14	0.977
DASH 15	0.977
DASH 16	0.977
DASH 17	0.977
DASH 18	0.977
DASH 19	0.977
DASH 20	0.978
DASH 21	0.978
DASH 22	0.977
DASH 23	0.977
DASH 24	0.978
DASH 25	0.977
DASH 26	0.978
DASH 27	0.977
DASH 28	0.977
DASH 29	0.978
DASH 30	0.978
Scale Reliability Statistics	
	Cronbach's α
Scale	0.978

Table S2. DASH activities subscale item reliability statistics (Cronbach's α=0.98)

	**If item dropped**
	**Cronbach's α**
DASH 1	0.974
DASH 2	0.975
DASH 3	0.974
DASH 4	0.974
DASH 5	0.974
DASH 6	0.975
DASH 7	0.974
DASH 8	0.975
DASH 9	0.975
DASH 10	0.974
DASH 11	0.974
DASH 12	0.974
DASH 13	0.975
DASH 14	0.975
DASH 15	0.975
DASH 16	0.974
DASH 17	0.975
DASH 18	0.974
DASH 19	0.975
DASH 20	0.976
DASH 21	0.976
DASH 22	0.975
DASH 23	0.975
Scale Reliability Statistics	
	Cronbach's α
Scale	0.976

Table S3. DASH symptoms item reliability statistics (Cronbach's α=0.92)

	**If item dropped**
	**Cronbach's α**
DASH 24	0.906
DASH 25	0.903
DASH 26	0.914
DASH 27	0.902
DASH 28	0.902
DASH 29	0.910
Scale Reliability Statistics	
	Cronbach's α
Scale	0.919

Table S4. Quick DASH item reliability statistics (Cronbach's α=0.94)

	If item dropped
	**Cronbach's α**
DASH 1	0.933
DASH 7	0.932
DASH 10	0.934
DASH 14	0.937
DASH 16	0.934
DASH 17	0.934
DASH 22	0.932
DASH 23	0.932
DASH 24	0.937
DASH 26	0.940
DASH 29	0.938
Scale Reliability Statistics	
	Cronbach's α
Scale	0.941

Table S5. PRWE item reliability statistics (Cronbach's α=0.97)

	If item dropped
	**Cronbach's α**
PRWE 1	0.966
PRWE 2	0.964
PRWE 3	0.964
PRWE 4	0.965
PRWE 5	0.965
PRWE 6	0.962
PRWE 7	0.962
PRWE 8	0.962
PRWE 9	0.962
PRWE 10	0.963
PRWE 11	0.963
PRWE 12	0.963
PRWE 13	0.962
PRWE 14	0.963
PRWE 15	0.963
Scale Reliability Statistics	
	Cronbach's α
Scale	0.965

Table S6. PRWE factor 1 - function item reliability statistics (Cronbach's α=0.97)

	If item dropped
	**Cronbach's α**
PRWE 6	0.963
PRWE 7	0.964
PRWE 8	0.963
PRWE 9	0.964
PRWE 10	0.965
PRWE 11	0.965
PRWE 12	0.965
PRWE 13	0.963

PRWE 14	0.965
PRWE 15	0.964
Scale Reliability Statistics	
	Cronbach's α
Scale	0.968

Table S7. PRWE factor 2 - pain item reliability statistics (Cronbach's α=0.97)

	If item dropped
	Cronbach's α
PRWE 1	0.926
PRWE 2	0.894
PRWE 3	0.907
PRWE 4	0.907
PRWE 5	0.911
Scale Reliability Statistics	
	Cronbach's α
Scale	0.926

Table S8. MWS item reliability statistics (Cronbach's α=0.71)

		If item dropped
	Item-rest correlation	Cronbach's α
MWS 1	0.423	0.694
MWS 2	0.452	0.677
MWS 3	0.557	0.613
MWS 5	0.567	0.607
Scale Reliability Statistics		
		Cronbach's α
Scale		0.712

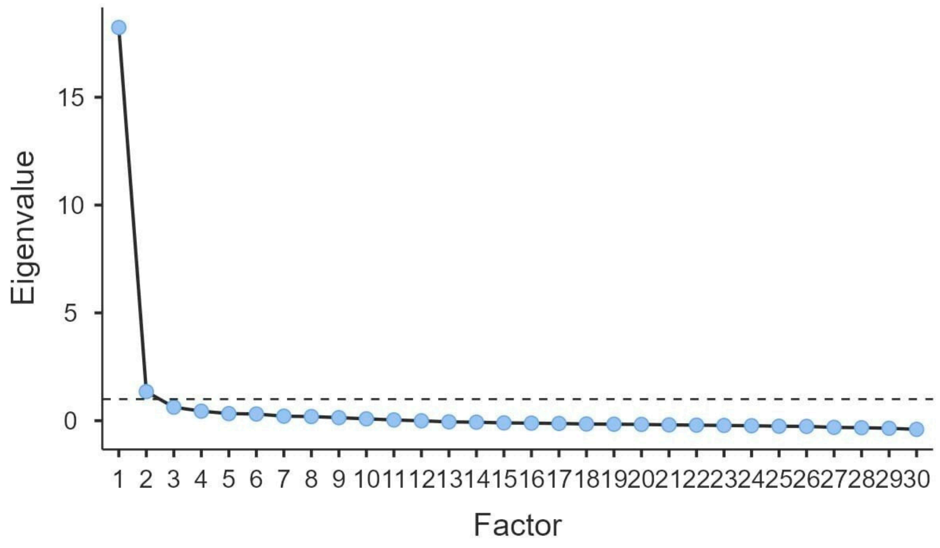

Figure S1. Scree plot of the DASH factor analysis.

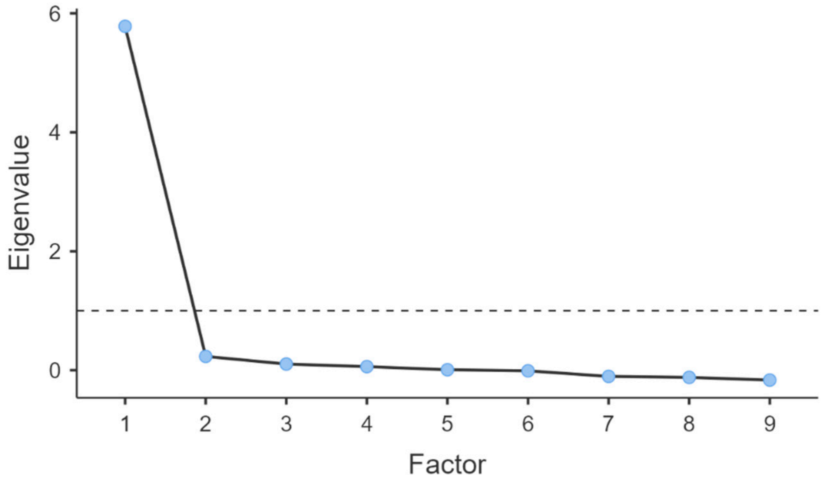

Figure S2. Scree plot of the quick-DASH factor analysis

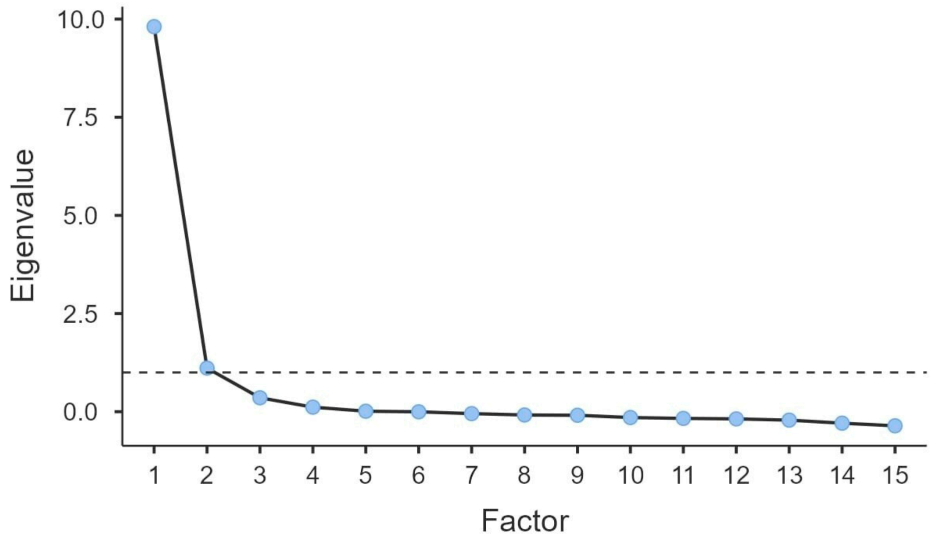

Figure S3. Scree plot of the PRWE factor analysis.

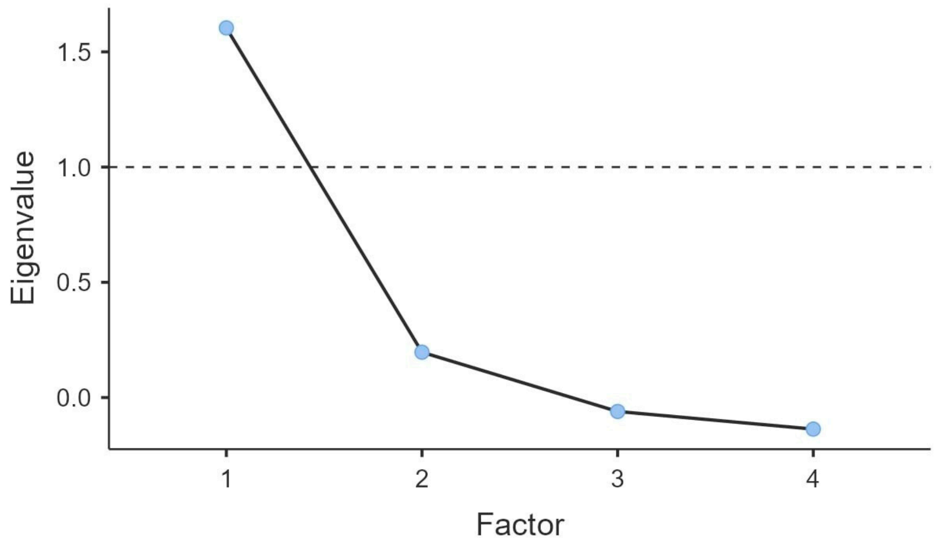

Figure S4. Scree plot of the MWS factor analysis.

Table S9. Structure matrices (correlations of each item with the extracted dimensions) of the quick DASH factor analysis

	**Factor**
	**1**
DASH 1	0.823
DASH 7	0.864
DASH 10	0.794
DASH 14	0.740
DASH 16	0.808
DASH 17	0.802
DASH 22	0.835
DASH 23	0.846
DASH 24	0.687
Eigen	5.78
% variance	64.2
